# Risk factors for incident delirium among older people in acute hospital medical units: a systematic review and meta-analysis

**DOI:** 10.1093/ageing/afu022

**Published:** 2014-03-06

**Authors:** Suman Ahmed, Baptiste Leurent, Elizabeth L. Sampson

**Affiliations:** 1 Tees, Esk and Wear Valleys NHS Foundation Trust, Durham DL2 2TS, UK; 2 Marie Curie Palliative Care Research Unit, Division of Psychiatry, University College London, London, UK; 3 Liaison Psychiatry, Barnet, Enfield and Haringey Mental Health Trust, London, UK

**Keywords:** delirium, risk factors, older people, hospitalised, medical unit

## Abstract

**Background**: delirium affects up to 40% of older hospitalised patients, but there has been no systematic review focussing on risk factors for incident delirium in older medical inpatients. We aimed to synthesise data on risk factors for incident delirium and where possible conduct meta-analysis of these.

**Methods**: PubMed and Web of Science databases were searched (January 1987–August 2013). Studies were quality rated using the Newcastle-Ottawa Scale. We used the Mantel–Haenszel and inverse variance method to estimate the pooled odds ratio (OR) or mean difference for individual risk factors.

**Results**: eleven articles met inclusion criteria and were included for review. Total study population 2338 (411 patients with delirium/1927 controls). The commonest factors significantly associated with delirium were dementia, older age, co-morbid illness, severity of medical illness, infection, ‘high-risk’ medication use, diminished activities of daily living, immobility, sensory impairment, urinary catheterisation, urea and electrolyte imbalance and malnutrition. In pooled analyses, dementia (OR 6.62; 95% CI (confidence interval) 4.30, 10.19), illness severity (APACHE II) (MD (mean difference) 3.91; 95% CI 2.22, 5.59), visual impairment (OR 1.89; 95% CI 1.03, 3.47), urinary catheterisation (OR 3.16; 95% CI 1.26, 7.92), low albumin level (MD −3.14; 95% CI −5.99, −0.29) and length of hospital stay (OR 4.85; 95% CI 2.20, 7.50) were statistically significantly associated with delirium.

**Conclusion**: we identified risk factors consistently associated with incident delirium following admission. These factors help to highlight older acute medical inpatients at risk of developing delirium during their hospital stay.

## Introduction

Delirium is a complex neuropsychiatric syndrome characterised by acute onset of disturbance of consciousness and fluctuating changes in cognition, attention and perceptual disturbance [1]. It is the most common reason for acute cognitive dysfunction in hospitalised older people. Prevalence of delirium at admission ranges from 10 to 31%, incidence of new delirium per admission ranges from 3 to 29% and occurrence rate per admission varies between 11 and 42% [2]. Delirium may be prevented in up to a third of older patients [3]; hence, early recognition is vital.

The UK National Institute for Health and Care Excellence (NICE) suggests screening for possible delirium based on four risk factors: age 65 or over, dementia, presentation with hip fracture and severity of illness [4]. However, these recommendations were developed from studies of a wide range of clinical populations recruited from surgical, intensive care and general medical settings. It is important to recognise that delirium risk factors may differ between medical and surgical patients where the latter are exposed to iatrogenic factors such as anaesthetic agents or surgical procedures. In addition, the NICE guidance includes studies where delirium was prevalent at baseline, did not use meta-analysis to identify key risk factors and focussed on ‘non-modifiable’ risk factors. Other predictive models for delirium in older people with general medical admission include a wider range of factors such as malnutrition, use of a urinary catheter and physical restraints [5].

There has been one previous systematic review of risk factors for delirium [6] but this considered older people admitted to a range of medical and surgical specialties and did not separate prevalent delirium (present on hospital admission) and incident delirium (that which occurs during the course of admission).

No systematic review has specifically evaluated risk factors for incident delirium in elderly hospitalised medical inpatients. Given that delirium is associated with poor outcomes including prolonged hospital stay, diminished cognitive and physical functioning, increased institutionalisation and a higher mortality risk [2], identifying robust delirium risk factors, particularly focussing on incident cases (which may be preventable) in older medical inpatients may improve the detection of delirium and improved targeting of interventions.

### Aim

The primary aim of this systematic review and meta-analysis was to identify risk factors for incident delirium in older people admitted to acute hospital medical units and to estimate the pooled odds ratio (OR) or mean difference (MD) of the reported risk factors. A secondary aim was to examine the scope, methodology and quality of the literature.

## Methods

### Search strategy

We used the MeSH terms ‘Confusion’ and ‘Causality’ to search PubMed. The MeSH (Medical Subject Headings) thesaurus defines the term ‘Confusion’ to include delirium, confusional state, disorientation and post-ictal confusion. The term ‘Causality’ includes risk factor, predisposing factor, precipitating factor, causation and reinforcing factor. We also searched using free text keywords ‘Delirium’ and ‘Risk factors’.

The above searches were repeated in ISI Web of Science. Search terms were kept broad to identify as many relevant publications as possible. We searched the databases for the period between 1 January 1987 and 31 August 2013 as this covers the time during which validated delirium assessment tools such as the DSM-III [7], Delirum Rating Scale (DRS) [8], NEECHAM scale [9] and CAM [10] have been used. We hand searched the reference lists of key journals in the field, previous review articles of risk factors for delirium, and also the citation lists of all included studies. Two authors reviewed titles of papers (S.A. and E.L.S.) and identified abstracts for further inspection. Where there was disagreement an independent adjudicator (a specialist systematic reviewer) made the decision on inclusion.

### Inclusion criteria

- Humans aged 55 years and over
- Published in English
- Primary research evaluating risk factors for incident delirium only
- Validated tools or criteria used to identify delirium
- Cohort, case–control and cross-sectional studies
- Admitted to medical/geriatric settings or acute medical settings.

### Exclusion criteria

- Studies of delirium tremens: this is a discrete condition with different underlying pathophysiological cause
- Studies conducted in intensive care units were excluded as these patients are exposed to a different range of pharmacological and environmental risks.

### Quality assessment

Two researchers (S.A. and E.L.S.) independently assessed the methodological strength of included studies to aid interpretation the validity of any findings using the Newcastle-Ottawa Scale (NOS) [11]. This was developed to assess the quality of design of non-randomised studies and consists of eight items, divided into three broad criteria: selection, comparability and-depending on the study type-outcome (cohort studies) or exposure (case–control studies). Studies are awarded a maximum of one star for each item with the exception of the item related to comparability that allows the assignment of two stars. Scores range between zero and nine stars (highest quality).

### Risk factor analysis

Selected articles were evaluated using a standardised checklist to identify all risk factors studied. This was developed using factors listed in the NICE Delirium guidelines and previous review articles. We wished to capture the widest possible range of risk factors studied so we iteratively adapted the checklist as we reviewed papers; if we found a risk factor that we had not previously identified this was added. We examined the reported statistics (OR, hazard ratio, relative risk, *P*-values and 95% CI) described in univariable analysis to determine the direction of association of a particular risk factor and whether it was statistically significant. Risk factors were tabulated as either ‘plus (+)’ ‘zero (0)’ or ‘minus (−)’, a plus sign indicating a factor which increases delirium risk, a minus sign indicates a protective factor and a zero indicating no statistically significant association with delirium risk. ‘Independent’ risk factors were identified from studies where multivariable analyses were conducted.

### Statistical methods

Where two or more studies examined a risk factor using a consistent measure and data were given as either numbers/counts (categorical data) or mean/SD (continuous data) and there was adequate information on numbers of case and control subjects we conducted meta-analysis. Numbers and types of medication were diversely measured and reported. It was therefore not possible to pool data on single medication classes such as neuroleptics or opioids. We therefore present these results by individual study and single drug class, considering ‘high-risk’ medications to include those identified in NICE delirium guidelines; sedatives, benzodiazepines, opiates, H2 receptor antagonists, neuroleptics, antiepileptics, antidepressants and anti-cholinergic drugs. Some studies calculated the mean number of medications and defined this as ‘polypharmacy’. Age, APACHE II (Acute Physiology and Chronic Health Evaluation scale) scores, polypharmacy, albumin level and length of hospital stay were treated as continuous variables. Sex, dementia, visual impairment and having a urinary catheter were treated as categorical variables.

We estimated the pooled OR for categorical data using the Mantel–Haenszel estimator and pooled mean difference for continuous data using the inverse variance method in Review Manager (Version 5.1, The Cochrane Collaboration, 2011). We used a random-effects model when statistical heterogeneity was present (*I*² ≥ 50%), and a fixed effect model in the absence of statistically significant heterogeneity.

## Results

**Figure 1.**
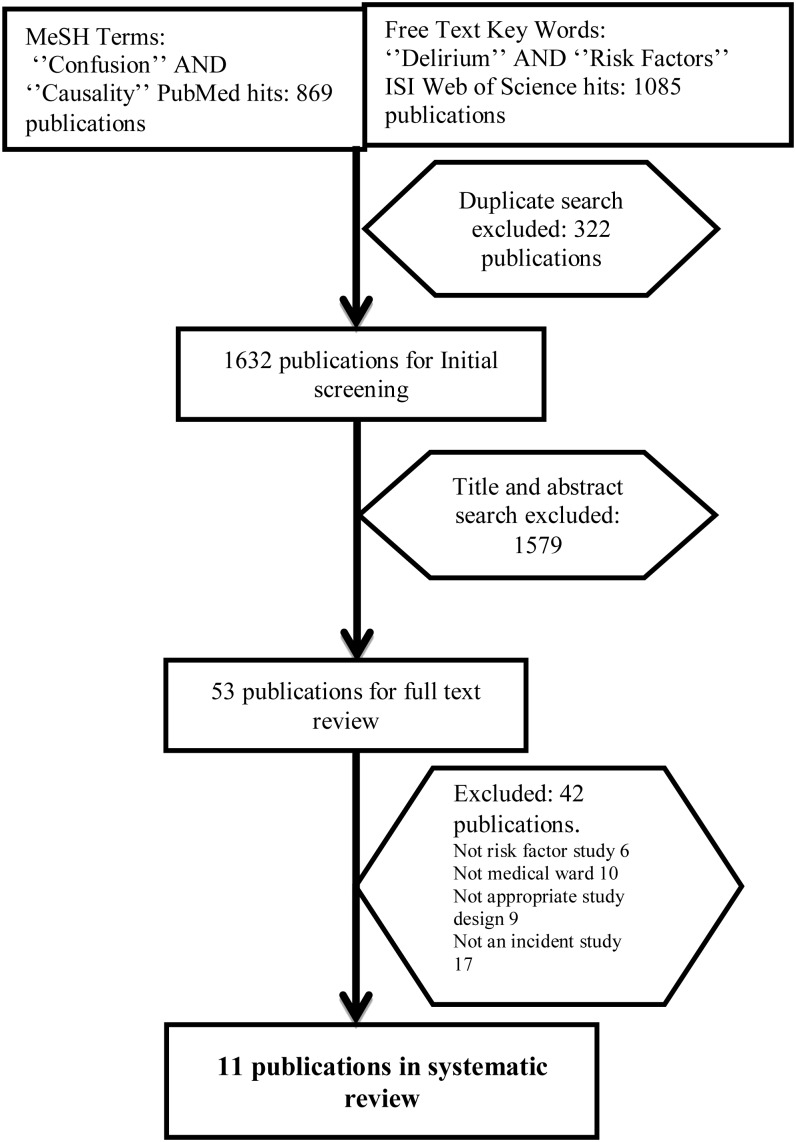
Selection of studies for review.

After removing duplicates, a total of 1,632 articles remained. After initial abstract and title screening, 53 articles were fully reviewed and 11 met inclusion criteria (nine cohort and two case–control studies of incident delirium). A total of 2,338 subjects were studied (411 delirium cases and 1,927 non-delirious controls) (Figure 1).

The mean age of participants ranged from 73 to 84.5 years (Table 1). All studies reported gender (total 2,338 participants); 1,177 (50.34%) were male and 1,161 (49.66%) were female. Tools used to identify delirium were the Diagnostic and Statistical Manual of Mental Disorders (4th and 3rd edition)-DSM-III [12], Confusion Assessment Method—CAM [10], Delirium Rating Scale—DRS [8], Delirium Assessment Scale—DAS [13], the NEECHAM Confusion Scale [9], Mini-Mental State Examination (MMSE) [14] and Clinical Assessment of Confusion Scale (CAC) [15]. Studies were conducted in United States (four studies), United Kingdom (two studies), Italy (two studies) and one each from Columbia, Mexico and Australia.

**Table 1.** Characteristics of studies examining risk factors for incident delirium in older acute medical inpatients

Study (authors, publication year, country)	Study design	Study setting	Sample size: case/control	Age: mean (SD), range	Sex: male/female	Type of cases: I/P (%)	Criteria for delirium	Assessment frequency (h)	Study quality (star)^a^
Franco *et al.*, 2010, Colombia [16]	Case Control	Medical	34/257	74.4 (8.79), 60–99	105/186	I (11.7)	CAM, DRS	24	9
Bo Mario *et al.*, 2009, Italy [17]	Cohort	Medical/Geriatric	28/224	82.4 (4.1)	121/131	I (11.1)	CAM, DRS	48	9
Ranhoff *et al.*, 2006, Italy [18]	Cohort	SICU	117/284	78.1 (8.8)	205/196	I (16.2), P (15.5)	CAM	24	8
Wilson *et al.*, 2005, United Kingdom [19]	Cohort	Medical	12/88	84.5 (4.2)	69/31	I (12)	CAM, DSM-III	24	8
Villalpando-Berumen *et al.*, 2003, Mexico [20]	Case Control	Medical	66/219	NA	130/155	I (12)	CAM	24	9
Wakefield, 2002, United States [21]	Cohort	Medical	16/101	73 (4.6), 65–89	117/0	I (14)	NEECHAM	24	7
Inouye *et al.*, 1996, United States [5]	Cohort	Medical	35/161	78.5 (5.7)	84/112	I (18)	CAM	48	9
O'Keeffe *et al.*, 1996, United Kingdom [22]	Cohort	Geriatric	28/72	82.7 (6.2)	37/63	I (28)	DSM-III, DAS	48	7
Foy *et al.*, 1995, Australia [23]	Cohort	Medical	21/397	70.2 (6.8), 60–88	234/184	I (5)	DSM–III-R	48–72	7
Inouye *et al.*, 1993, United States [24]	Cohort	Medical	27/80	79.3 (6.6)	49/58	I (25)	CAM	24	8
Foreman Marquis, 1989, United States [25]	Cohort	Medical	27/44	73.63 (7.65)	26/45	I (38)	MMSE, CAC	24	6

^a^Quality assessment by NOS.

CAM, Confusion Assessment Method; DRS, Delirium Rating Scale; DSM–III-R, Diagnostic and Statistical Manual of Mental Disorders (3rd Ed-R), NEECHAM Confusion Scale; DAS, Delirium Assessment Scale; MMSE, Mini-Mental State Examination; CAC, Clinical Assessment of Confusion; I, Incident; P, Prevalent; SICU, sub intensive care unit.

### Study quality

Quality scores ranged from 6 to 9 stars (median 8 stars) (Table 1). Most of the studies (9 out of 11) scored maximum points (four stars) in ‘study selection’ criteria. In comparability of cohorts/cases and controls criteria 7 out of 11 studies scored maximum points (two stars). In ‘outcome’ criteria, only five studies achieved the maximum three stars.

### Incidence of delirium in included studies

The incidence of delirium ranged between 5 and 38% [5, 16–25]. In seven studies [16, 18–21, 24, 25], delirium assessment was undertaken at 24 h intervals during the admission.

### Risk factors

We identified 49 risk factors studied in univariable analysis. Of these, 29 were studied in two or more studies (Supplementary data are available in *Age and Ageing online*, Appendix 1). The remaining 20 factors were included in only one study and are mentioned briefly in the text. Seven studies reported 20 ‘independent’ risk factors in multivariable analysis (Table 2).

**Table 2.** Multivariable analyses of risk factors for incident delirium in older medical inpatients

Risk factors	OR*/RR** (95% CI)	Study
Mental status		
Dementia	2.06** (1.62–2.64)	Bo *et al.* [17]
	2.82** (1.19–6.65)	Inouye *et al.* [24]
	3.26* (1.18–9.04)	Wilson *et al.* [19]
Depression	8.99* (1.59–50.76)	Wilson *et al.* [19]
Physical illness		
Illness severity	1.29** (1.11–1.51)	Bo *et al.* [17]
	3.49** (1.48–8.23)	Inouye *et al.* [24]
Co-morbidity	1.16* (1.04–1.30)	Villalpando-Berumen *et al.* [20]
Medication		
Polypharmacy	2.9** (1.6–5.4)	Inouye *et al.* [5]
	1.9* (1.1–3.2)	Ranhoff *et al.* [18]
Physical status		
Diminished ADL	8.4* (1.1–62.1)	Wakefield [21]
Urinary catheter	2.4** (1.2–4.7)	Inouye *et al.* [5]
	2.7* (1.4–4.9)	Ranhoff *et al.* [18]
Physical restraints	4.4** (2.5–7.9)	Inouye *et al.* [5]
Visual impairment	3.51** (1.15–10.71)	Inouye *et al.* [24]
Laboratory findings		
Malnutrition/low albumin	4.0** (2.2–7.4)	Inouye *et al.* [5]
	0.50* (0.26–0.95)	Villalpando-Berumen *et al.* [20]
	10.7* (1.5–74.5)	Wakefield [21]
Azotemia/Urea Abnormal	2.02** (0.89–4.60)	Inouye *et al.* [24]
Leucocyte abnormal	0.44* (0.21–0.90)	Villalpando-Berumen *et al.* [20]
Low haematocrit	2.16* (1.01–4.60)	Villalpando-Berumen *et al.* [20]
IGF-1	0.82* (0.69–0.97)	Wilson *et al.* [19]
Miscellaneous		
Iatrogenic events	1.9** (1.1–3.2)	Inouye *et al.* [5]
Stressful event	3.36** (2.86–5.44)	Bo *et al.* [17]
Heavy Alcohol use	6.1* (1.8–19.6)	Ranhoff *et al.* [28]
Prolonged hospital stay	1.07* (1.02–1.11)	Villalpando-Berumen *et al.* [20]
Smoking	0.2* (0.03–1.1)	Wakefield [21]

*results reported as odds ratio (OR).

**results reported as risk ratio (RR).

We were able to estimate the pooled OR (categorical outcomes) or mean difference (continuous outcomes) on nine risk factors (Table 3). We observed greater heterogeneity in some risk factors; old age, illness severity (APACHE II), length of hospitalisation, low albumin, visual impairment and urinary catheterisation and less heterogeneity for male sex, dementia and polypharmacy (*for forest plots, see* supplementary data in *Age and Ageing* online, Appendix 2, Figure 1–9).

**Table 3.** Meta-analysis of risk factors for incident delirium in older medical inpatients

Risk factor	Studies/total sample (*n*/*n*)	Statistical method	Pooled OR or MD* (95% CI)	Heterogeneity*I*^2^ (%)
Demographic factors				
Old age	5/1,300	IV, Random	2.74 [0.11, 5.38]*	86
Male sex	5/1,148	M–H, Fixed	0.86 [0.65, 1.14]	0
Mental status				
Dementia	2/501	M–H, Fixed	6.62 [4.30, 10.19]	0
Physical illness				
Illness severity (APACHE II)	2/653	IV, Random	3.91 [2.22, 5.59]*	69
Physical status				
Visual impairment	4/1,077	M–H, Random	1.89 [1.03, 3.47]	64
Urinary catheterisation	2/692	M–H, Random	3.93 [2.51, 6.14]	62%
Medication				
Polypharmacy	3/944	IV, Fixed	0.64 [0.17, 1.11]*	0
Laboratory findings				
Low albumin	2/518	IV, Random	−3.14 [−5.99, −0.29]*	68
Hospitalisation related				
Length of hospital stay	2/537	IV, Random	4.85 [2.20, 7.50]	69

OR, odds ratio; MD, mean difference; CI, confidence interval; M–H, Mantel–Haenszel method; IV, inverse variance method.

*indicates that mean difference is reported.

### Demographic factors

Age was the most frequently studied risk factor (nine studies). Four studies [16, 18, 20, 21] reported old age as statistically significantly associated with increased delirium risk in pooled analysis; mean difference 2.74 (95% CI 0.11, 5.38, *P* = 0.04). Male sex was not significantly associated with delirium risk in pooled analysis.

### Mental status

There was considerable variability in defining dementia. Most studies (six out of seven) [5, 16, 18, 21, 22, 24] used the MMSE, and one study [19] used The Informant Questionnaire on Cognitive Decline in the Elderly (IQCODE) in addition to MMSE. Dementia was significantly associated with delirium in six studies [16–19, 21, 22, 24]. This association remained significant in three multivariable analyses [17, 19, 24] and was statistically significant in pooled analysis (OR 6.62, 95% CI 4.30, 10.19, *P* < 0.001). Depression was statistically significantly associated with increased delirium risk [19, 25] in two studies (univariable) and in one multivariable analysis [19].

### Physical illness

Illness severity was measured by the Acute Physiology and Chronic Health E valuation (APACHE II) scale in most studies (four out of five) [17–19, 24]. Most studies consistently reported ‘illness severity’ [17, 18, 22, 24] and ‘co-morbidity’ [17, 18, 20] as significant risk factors in univariable and multivariable analyses [17, 18, 24]. Pooled analysis was statistically significant for the mean APACHE II score (MD 3.91, 95% CI 2.22, 5.59), *P* < 0.001). Two studies [16, 21] reported infection/UTI as a statistically significant risk factor in univariable analysis.

### Activities of daily living, vision and hearing

Diminished ADL skills [17, 18, 21], immobility [5, 21] and urinary catheters [5, 18] were statistically significant delirium risk factors. One study [21] reported diminished ADL skills as a significant independent risk factor. Urinary catheterisation [5, 18] was a statistically significant independent risk factors in two studies and in pooled analysis (OR 3.16, 95% CI 3.16, 1.26, 7.92, *P* = 0.01). The evidence for visual [17, 18, 20, 22, 24] and auditory impairment [17, 24] was inconclusive in univariable analysis; however, combined odds of developing delirium for visual impairment was significant (OR 1.89, 95% CI 1.03, 3.47, *P* = 0.04).

### Medication

Use of ‘high-risk medications’ such as narcotics, major tranquilisers [5], neuropleptics, narcotics and benzodiazepines [22] was not associated with delirium in univariable analyses. Benzodiazepines at daily equivalent dose of 5 mg or more per day gave a statistically significant increase in delirium risk in adjusted analysis (OR 3.5, 95% CI 1.4–8.8) and being on neuroleptics or benzodiazepines *on admission* was associated with delirium in univariable analysis [17]. Four of six studies [5, 18, 19, 25] reported polypharmacy as a significant delirium risk factor in univariable analysis, this was confirmed in two multivariable analyses [5, 18] and was statistically significant in pooled analysis (MD 0.64 95% CI 0.17, 1.11, *P* = 0.008).

### Laboratory investigations

Low albumin [5, 18, 21], high or low sodium [21, 22, 25] and urea/creatinine ratio abnormality [18, 21, 24, 25] were most commonly associated with increased delirium risk. In pooled analysis, low albumin was statistically significantly associated with delirium (MD −3.14, 95% CI −5.99, −0.29, *P* = 0.03). Insulin-like growth factor, IGF-1 was found as a protective factor for delirium in one study [19] in multivariable analysis. Low haematocrit was significant in univariable analysis in two studies [18, 21] and multivariable analysis in one study [20]. High and low glucose level [18, 20] showed inconsistent association with increased delirium risk.

### Hospital-related factors

Increased length of hospital stay was strongly associated with delirium in univariable [17, 20], one multivariable [20] and pooled analyses (MD –days 4.85, 95% CI 2.20, 7.50, *P* < 0.001).

### Miscellaneous factors

One univariable [18] and one multivariable [18] study reported a significant association with excess alcohol use. Iatrogenic [5] and stressful life events [17] were independent delirium risk factors in one multivariable analysis each.

A total of 20 risk factors were reported in only one study. Recent stressful events [17], geriatric acute care [17], any iatrogenic events [5], low blood pressure [25] and low or high potassium level [25] were significantly associated with delirium. Other factors that did not show a significant association with delirium included marital and occupational status [16], living alone [17], mechanical ventilation [18], mean cell volume [20], abnormal temperature [22] and recent surgical procedure [17], other medication use [22], low or high urea level [22], low body mass index and cholesterol level [20], ethnic origin [25] and urinary elimination problem [23]. Two studies reported one protective factor each: IGF-1 [19] and smoking [21].

## Discussion

We identified 11 studies which investigated risk factors for incident delirium in older people with acute medical admission. Of the risk factors examined, 10 consistently showed statistically significant association with incident delirium in both univariable and multivariable analysis: dementia, co-morbid physical illness, severity of physical illness (as measured by APACHE II) , poor ADL function, urinary catheterisation, polypharmacy, low albumin, urea/creatinine ratio abnormality (azotemia), low or high sodium and prolonged hospital stay. Our pooled analysis confirmed statistically significant associations for dementia, illness severity (mean APACHE II score), urinary catheterisation, polypharmacy, albumin level and length of hospital stay.

Previous meta-analysis of studies from mixed hospital settings showed male gender, depression and abnormal sodium level as significant risk factors but our analysis did not replicate those findings [6]. This may be because we focussed on incident cases only and studies conducted in older medical inpatients.

### Methodological quality of the included studies

We used a validated tool, the NOS to evaluate the quality of the studies. A high level of agreement was found between the two reviewers and overall study quality was good. In general, controls were recruited from the same population as cases and cases were identified using validated tools such as the CAM. The studies were conducted in a wide range of countries, which may offer more generalisability when combining data. The number of participants in the included publications ranges from 71 to 418 patients. The smaller studies [19, 22, 25] may be underpowered for finding statistically significant risk factors.

Most of the studies used the validated diagnostic algorithm of the CAM to diagnose delirium except four studies which used the DSM-III [19, 22], DSM-III-R [23] DAS and NEECHAM confusion scale [21] and CAC [25].

### Strengths and limitations of this review

We have been able to pool data from over 400 cases of incident delirium and have, as recommended by previous authors focussed on a single hospital service, older medical inpatients [6]. Our review was limited to articles published in English which may have resulted in the exclusion of relevant studies in non-English speaking countries. We assessed the methodological quality of the included studies with NOS which revealed satisfactory quality though all studies lost one star in ‘comparability’ section. Tabulating results was challenging because of differences in the definitions, measurement and statistical analysis of some risk factors. For example, there was no standardised definition of ‘old age’. Heterogeneity was particularly marked in some risk factors, for example, old age, comorbid illness, length of hospitalisation and sodium level and low albumin; hence, we used a random-effects model for them in pooled analysis. Few studies explicitly stated the variables that were adjusted for in multivariable analysis. Estimation of the ‘independent’ effect of each factor depends on which variables it was adjusted on, and caution is required when interpreting estimates in the absence of more information. We did not include data from randomised controlled trials of delirium interventions as trial participants may be atypical compared with the wider population of people with delirium.

### Implications for research

Risk factors for development of delirium are diverse and how they interact needs to be explored further. For example, dementia is a well-established independent predictor of delirium, consistently demonstrated in many studies including our meta-analysis but the underlying pathophysiology of this association is not well understood. Studies used a range of methods to diagnose dementia and in future this should be standardised to widely accepted clinical criteria such as DSM-IV.

In addition, only limited inference can be made about the causal nature of the associations. For example, the significance of prolonged hospital stay in relation to delirium may be explained in a number of ways: lengthy hospitalisation itself may increase the time at risk to develop delirium, or length of stay may be associated with comorbidities such as dementia, which in themselves predispose to delirium. Other factors such as frailty, which is a marker for a range of poor outcomes in older hospital medical inpatients [26], were not explored in these studies. Definitions of frailty are becoming more clearly operationalised and future studies of risk factors for delirium should consider the use of standardised frailty measures.

Assessing the level of acute illness on hospital admission is important; however, the studies we identified used the APACHE II scale. Future studies should consider using systems which are widely in day-to-day clinical practice such as versions of the National Early Warning Score [27]. This would aid in the interpretation of research results and their translation into clinical practice. Given the number and complexity of potential confounding factors in the association between risk factors and delirium, we were surprised to find how few studies conducted controlled analyses. Where this was done, papers rarely explicitly stated which variables were adjusted for. Future research should be adequately powered and ensure that all variables are carefully described in adjusted analyses.

Researching incident delirium is challenging and this may be why we identified so few studies that consider risk factors for this. By definition, symptoms of delirium can be fleeting and vary over time. This requires that patients have regular detailed clinical examination, ideally at least once every 24 h and this has significant implications for staffing of research teams and the costs of studies.

### Implications for clinical practice

Despite these research challenges, our meta-analysis found certain risk factors to be consistently associated with incident delirium. Some of these may be modifiable, for example, medical illness related factors, laboratory abnormalities such as low albumin and polypharmacy. Others are non-modifiable, for example, age, gender and dementia but are still clinically useful in highlighting which patients are most at risk. These findings give strength to existing predictive risk models of delirium [5, 24].

Risk factors such as age, dementia and severity of illness are identified in the NICE Delirium guidance; however, in addition, our findings highlight that in an older person undergoing acute medical admission, polypharmacy, poor vision, low albumin and having a urinary catheter indicate vulnerability to developing delirium. Management of these potentially modifiable factors has been found to be integral to successful multicomponent interventions for delirium in older people [3, 28].

**Key points**

- Delirium is common in older adults hospitalised due to acute medical conditions with incidence during admission of 5–38%.
- Significant risks in meta-analysis were age, dementia, severe illness, poor vision, urinary catheters, polypharmacy and low albumin.
- These potentially modifiable factors could be included in multicomponent delirium interventions in this group of patients.

## Supplementary data

Supplementary data mentioned in the text are available to subscribers in *Age and Ageing* online.

## Conflicts of interest

none declared.

## Supplementary Material

Supplementary Data

## References

[1] American Psychiatric Association (2000). Diagnostic and Statistical Manual of Mental Disorders: DSM-IV-TR.

[2] Siddiqi N, House AO, Holmes JD (2006). Occurrence and outcome of delirium in medical in-patients: a systematic literature review. Age Ageing.

[3] Inouye SK, Bogardus ST, Charpentier PA (1999). A multicomponent intervention to prevent delirium in hospitalized older patients. N Engl J Med.

[4] Young J, Murthy L, Westby M, Akunne A, O'Mahony R (2010). Diagnosis, prevention, and management of delirium: summary of NICE guidance. BMJ.

[5] Inouye SK, Charpentier PA (1996). Precipitating factors for delirium in hospitalized elderly persons. Predictive model and interrelationship with baseline vulnerability. JAMA.

[6] Elie M, Cole MG, Primeau FJ, Bellavance F (1998). Delirium risk factors in elderly hospitalized patients. J Gen Intern Med.

[7] American Psychiatric Association (1980). Diagnostic and Statistical Manual-III.

[8] Trzepacz PT, Mittal D, Torres R, Kanary K, Norton J, Jimerson N (2001). Validation of the Delirium Rating Scale-revised-98: comparison with the delirium rating scale and the cognitive test for delirium. J Neuropsychiatry Clin Neurosci.

[9] Neelon VJ, Champagne MT, Carlson JR, Funk SG (1996). The NEECHAM Confusion Scale: construction, validation, and clinical testing. Nurs Res.

[10] Inouye SK, van Dyck CH, Alessi CA, Balkin S, Siegal AP, Horwitz RI (1990). Clarifying confusion: the confusion assessment method. A new method for detection of delirium. Ann Intern Med.

[11] Wells GA, Shea B, O'Connell D (2000). The Newcastle-Ottawa Scale (NOS) for assessing the quality of nonrandomised studies in meta-analyses.

[12] Johnson JC, Gottlieb GL, Sullivan E (1990). Using DSM-III criteria to diagnose delirium in elderly general medical patients. J Gerontol.

[13] O'keeffe ST (1994). Rating the severity of delirium: The delirium assessment scale. Int J Geriatr Psychiatry.

[14] Folstein MF, Folstein SE, McHugh PR (1975). ‘Mini-mental state’. A practical method for grading the cognitive state of patients for the clinician. J Psychiatr Res.

[15] Vermeersch PE (1990). The clinical assessment of confusion-A. Appl Nurs Res.

[16] Franco JG, Valencia C, Bernal C (2010). Relationship between cognitive status at admission and incident delirium in older medical inpatients. J Neuropsych Clin Neurosci.

[17] Bo M, Martini B, Ruatta C (2009). Geriatric ward hospitalization reduced incidence delirium among older medical inpatients. Am J Geriatr Psychiatry.

[18] Ranhoff AH, Rozzini R, Sabatini T, Cassinadri A, Boffelli S, Trabucchi M (2006). Delirium in a sub-intensive care unit for the elderly: occurrence and risk factors. Aging Clin Exp Res.

[19] Wilson K, Broadhurst C, Diver M, Jackson M, Mottram P (2005). Plasma insulin growth factor-1 and incident delirium in older people. Int J Geriatr Psychiatry.

[20] Villalpando-Berumen JM, Pineda-Colorado AM, Palacios P, Reyes-Guerrero J, Villa AR, Gutierrez-Robledo LM (2003). Incidence of delirium, risk factors, and long-term survival of elderly patients hospitalized in a medical specialty teaching hospital in Mexico City. Int Psychogeriatr.

[21] Wakefield BJ (2002). Risk for acute confusion on hospital admission. Clin Nurs Res.

[22] O'Keeffe ST, Lavan JN (1996). Predicting delirium in elderly patients: development and validation of a risk-stratification model. Age Ageing.

[23] Foy A, O'Connell D, Henry D, Kelly J, Cocking S, Halliday J (1995). Benzodiazepine use as a cause of cognitive impairment in elderly hospital inpatients. J Gerontol A Biol Sci Med Sci.

[24] Inouye SK, Viscoli CM, Horwitz RI, Hurst LD, Tinetti ME (1993). A predictive model for delirium in hospitalized elderly medical patients based on admission characteristics. Ann Intern Med.

[25] Foreman MD (1989). Confusion in the hospitalized elderly: incidence, onset, and associated factors. Res Nurs Health.

[26] Morley JE, Vellas B, van Kan GA (2013). Frailty consensus: a call to action. J Am Med Dir Assoc.

[27] Prytherch DR, Smith GB, Schmidt PE, Featherstone PI (2010). ViEWS – towards a national early warning score for detecting adult inpatient deterioration. Resuscitation.

[28] Inouye SK, Bogardus ST, Baker DI, Leo-Summers L, Cooney LM (2000). The Hospital Elder Life Program: a model of care to prevent cognitive and functional decline in older hospitalized patients. Hospital Elder Life Program. J Am Geriatr Soc.

