# Supplementary material for: Risk factors for incident delirium among older people in acute hospital medical units: a systematic review and meta-analysis
Source: Age Ageing. 2014 Mar 6;43(3):326–33. doi: 10.1093/ageing/afu022 (PMC4001175; doi:10.1093/ageing/afu022)
Supplement: Supplementary Data [file supp_43_3_326__index.html]

Risk factors for incident delirium among older people in acute hospital medical units: a systematic review and meta-analysis — Risk factors for incident delirium among older people in acute hospital medical units: a systematic review and meta-analysis — Supplementary Data 

# Risk factors for incident delirium among older people in acute hospital medical units: a systematic review and meta-analysis

## Supplementary Data

Supplementary Data

**Files in this Data Supplement:**

- Supplementary Data - Docx file
