# Supplementary material for: Risk factors for incident delirium among older people in acute hospital medical units: a systematic review and meta-analysis
Source: Age Ageing. 2014 Mar 6;43(3):326–33. doi: 10.1093/ageing/afu022 (PMC4001175; doi:10.1093/ageing/afu022)
Supplement: Supplementary Data [file supp_afu022_afu022supp.docx]

**Supplementary data-Appendix 1: Risk factors for incident delirium in older medical inpatients**

| **Risk Factor** | **Bo et al. 2009** | **Franco et al. 2010** | **Inouye et al. 1996** | **O'Keeffe et al. 1996** | **Ranhoff et al. 2006** | **Villalpando-Berumen et al. 2003** | **Wilson et al. 2005** | **Wakefield 2007** | **Foreman 1989** | **Foy et al 1995** | **Inouye et al. 1993** |
| --- | --- | --- | --- | --- | --- | --- | --- | --- | --- | --- | --- |
| **DEMOGRAPHIC FACTORS:** |  |  |  |  |  |  |  |  |  |  |  |
| Old age | **0** | **+** |  | **0** | **+** | **+** | **0** |  | **0** | **+** | **0** |
| Male sex | **0** | **0** |  | **0** | **0** | **0** | **0** |  | **0** |  | **0** |
| Level of education | **0** |  |  |  |  | **+** |  |  | **+** |  |  |
| **MENTAL STATUS** |  |  |  |  |  |  |  |  |  |  |  |
| Dementia | **+** | **+** |  | **+** | **+** |  | **+** | **+** |  |  | **+** |
| Past Delirium | **+** |  |  |  |  |  |  |  |  |  | **0** |
| Depression | **0** |  |  | **0** |  |  | **+** | **0** | **+** |  | **0** |
| **PHYSICAL ILLNESS** |  |  |  |  |  |  |  |  |  |  |  |
| Comorbidity | **+** |  |  |  | **+** | **+** |  |  |  |  |  |
| Medical illness |  | **0** |  |  |  | **0** |  |  | **0** | **+** |  |
| Illness severity (APACHE II) | **+** |  |  | **+** | **+** |  | **0** |  |  |  | **+** |
| Infection /UTI |  | **+** | **0** |  |  |  |  | **+** |  |  |  |
| **PHYSICAL STATUS** |  |  |  |  |  |  |  |  |  |  |  |
| Diminished ADL | **+** |  |  | **0** | **+** |  |  | **+** |  |  | **0** |
| Immobility |  |  | **+** |  |  |  |  | **+** |  |  |  |
| Visual impairment | **0** |  |  | **0** | **+** | **0** |  |  |  |  | **+** |
| Hearing impairment | **0** |  |  |  |  |  |  |  |  |  | **+** |
| Urinary catheter |  | **0** | **+** |  | **+** |  |  |  |  |  |  |
| Dehydration |  |  | **0** |  |  |  |  |  |  | **0** |  |
| Malnutrition | **0** |  | **+** |  |  |  |  |  |  |  |  |
| MEDICATION |  |  |  |  |  |  |  |  |  |  |  |
| Polypharmacy | **0** | **0** | **+** |  | **+** |  | **+** |  | **+** |  |  |
| High risk medications use* | **0** |  | **+/0** | **0** |  |  |  |  |  | **0** |  |
| **LABORATORY FINDINGS** |  |  |  |  |  |  |  |  |  |  |  |
| Abnormal sodium |  |  |  | **+** |  |  |  | **+** | **+** |  |  |
| Urea/Creatinine ratio |  |  |  |  | **+** |  |  | **+** | **+** |  | **+** |
| Abnormal plasma glucose |  |  |  |  | **0** | **+** |  |  |  |  |  |
| Low albumin |  |  | **+** | **0** | **+** | **0** |  | **+** |  |  |  |
| Low haematocrit |  |  |  |  |  | **+** |  | **+** |  |  |  |
| Abnormal leucocyte levels |  |  |  | **0** |  | **0** |  |  |  |  |  |
| **HOSPITALISATION RELATED** |  |  |  |  |  |  |  |  |  |  |  |
| Institutional living |  |  |  | **0** |  |  |  | **+** |  |  |  |
| Prolonged stay in emergency department | **0** |  | **0** |  |  |  |  |  |  |  |  |
| Length of hospital stay | **+** |  |  |  |  | **+** |  |  |  |  |  |
| **OTHERS** |  |  |  |  |  |  |  |  |  |  |  |
| Alcohol abuse |  |  |  | **0** | **+** |  |  |  |  |  |  |

Legend: A plus sign indicates a risk factor with statistical significance; a minus sign would indicate a protective factor with statistical significance, zero indicates the factor had failed to reach statistical significance of either type; blank cells indicate the factor was not studied.

*High risk medications includes- sedatives, benzodiazepines, opiates, and H2 receptor antagonists, neuroleptics, antiepileptics, antidepressants and anti-cholinergic drugs

**Supplementary data-Appendix 2 (figure 1-9): Forest plots of meta-analysis of risk factors for incident delirium in older medical inpatients**

**1. Old Age**

**2. Male sex**

**3. Dementia**

**4. Illness severity (APACHE II)**

**5. Visual impairment**

**6. Urinary catheterisation**

**7. Number of medications (Polypharmacy)**

**9. Length of hospitalisation**

**8. Mean Albumin level (g/l)***

*****Note: for mean albumin polarity of Forest plot is reversed
